# Cortical dysplasia and autistic trait severity in children with Tuberous Sclerosis Complex: a clinical epidemiological study

**DOI:** 10.1007/s00787-017-1066-z

**Published:** 2017-10-23

**Authors:** Sabine E. Mous, Iris E. Overwater, Rita Vidal Gato, Jorieke Duvekot, Leontine W. ten Hoopen, Maarten H. Lequin, Marie-Claire Y. de Wit, Gwendolyn C. Dieleman

**Affiliations:** 1 Department of Child and Adolescent Psychiatry/Psychology, Erasmus Medical Center-Sophia Children’s Hospital, P.O. Box 2060, 3000 CB Rotterdam, The Netherlands; 2 ENCORE Expertise Center for Neurodevelopmental Disorders, Erasmus Medical Center-Sophia Children’s Hospital, P.O. Box 2060, 3000 CB Rotterdam, The Netherlands; 3 Department of Pediatric Neurology, Erasmus Medical Center-Sophia Children’s Hospital, P.O. Box 2060, 3000 CB Rotterdam, The Netherlands; 4 Department of Radiology, University Medical Center Utrecht, P.O. Box 85500, 3508 GA Utrecht, The Netherlands

**Keywords:** Tubers, Radial migration lines, Autism, Quantitative autistic traits, Intelligence, Cognitive functioning

## Abstract

Tuberous Sclerosis Complex (TSC) is characterized by a high prevalence of autism spectrum disorders (ASD). Little is known about the relation between cortical dysplasia and ASD severity in TSC. We assessed ASD severity (using the Autism Diagnostic Observation Scale), tuber and radial migration line (RML) count and location, and cognitive functioning in 52 children with TSC and performed regression and mediation analyses. Tuber and RML count were strongly positively related to ASD severity. However, when correcting for cognitive functioning, the majority of associations became insignificant and only total tuber count remained associated to the severity of restricted/repetitive behaviors. Occipital RML count remained associated with overall ASD severity, and social communication/interaction deficit severity specifically. This study shows the important explanatory role of cognitive functioning in the association between cortical dysplasia and ASD severity, and the relevance of separately studying the two ASD subdomains.

**Electronic supplementary material:**

The online version of this article (doi:10.1007/s00787-017-1066-z) contains supplementary material, which is available to authorized users.

## Introduction

**Fig. 1:**
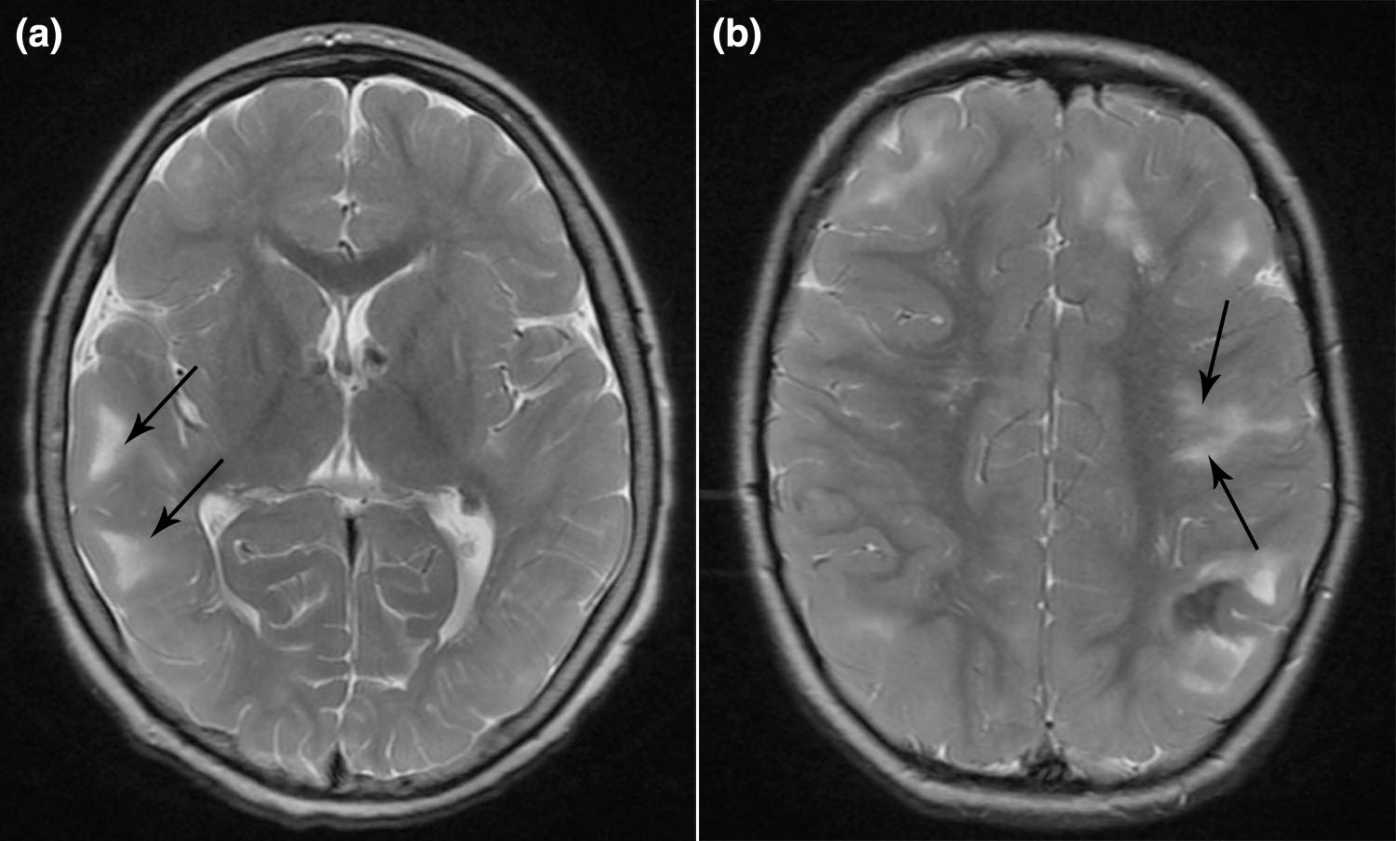
Example of T2-weighted images with arrows indicating **a** cortical tubers, and **b** radial migration lines

Tuberous Sclerosis Complex (TSC) is an autosomal dominant disorder affecting 1 in 6000 people, caused by inactivating *TSC1* (chromosome 9) or *TSC2* (chromosome 16) variants. The *TSC1* and *TSC2* protein products form the intracellular TSC1-TSC2 protein complex, which serves as a regulator of the mammalian target of rapamycin (mTOR) pathway. Mutations in the *TSC1* or *TSC2* gene lead to a upregulation of the mTOR pathway, causing uncontrolled cell growth and abnormal differentiation and the proliferation of benign overgrowths of cells and tissue in several organ systems including the brain, skin, kidneys, heart, eyes, lungs and bones [1]. In the brain this may lead to cortical dysplasia. The most common form of cortical dysplasia in TSC is the presence of cortical tubers, affecting over 80% of all TSC patients. Cortical tubers are focal developmental abnormalities of the cortex, characterized by disorganized lamination and atypical cellular growth, differentiation and maturation [2, 3], which develop during prenatal brain development and can be detected by MRI from 20 weeks of gestation onwards. Postnatally, no new tubers arise, but in older children tubers may calcify or become cystic [1]. Another form of cortical dysplasia is the presence of radial migration lines (RMLs). RMLs are linear abnormalities that extend from the ventricles to the cortex, representing areas of hypomyelination and white matter heterotopia [4]. RMLs are a marker of abnormal neural migration and cortical organization and are often associated with a tuber, but can also be isolated. Both forms of cortical dysplasia may act as epileptogenic lesions. In Fig. 1, an example of a Magnetic Resonance Imaging (MRI) scan showing cortical tubers and RMLs is provided.

Other features associated with brain pathology in TSC include the presence of epilepsy (72–85% of all patients) and cognitive impairment, with about 50% of patients having an intellectual disability (IQ < 70), and a range of behavioral and psychiatric symptoms. Autism spectrum disorder (ASD) is highly prevalent in children with TSC, with prevalence rates estimated around 40–50% [5–8].

Previous studies have suggested the total number of cortical tubers to be an important predictor for an ASD diagnosis in TSC, and the temporal lobes were suggested to be specifically implicated [9, 10]. Other studies found the presence (yes/no) of temporal tubers to be associated with a higher likelihood of an ASD diagnosis [11], while others specifically found the number of cyst-like tubers to be related to ASD diagnostic status [12] or found a diagnosis of autism to be related to frontal and posterior tubers [13]. Still others did not find an association between the occurrence of cortical tubers and an autism diagnosis [14], or found the number of tubers to be equally prevalent in intellectually disabled non-autistic and intellectually disabled autistic children and thus non-specific for ASD [15]. These inconsistencies in findings point out that the association between tuber burden and ASD is still poorly understood.

Although these previous studies have experimented with different ways of defining cortical tuber involvement (i.e. tuber presence (yes/no), tuber count, or tuber size), in these studies ASD has always only been categorically defined as the presence or absence of an ASD diagnosis. The notion that child psychopathology, such as ASD, might be better described within a quantitative—or dimensional—framework has gained support in the last years [16]. Within this framework of continuous symptom levels, the entire spectrum of symptom severity is covered. Most likely, this is a more naturalistic representation of psychopathology, as compared to the use of all-or-none dichotomous diagnostic categories. Previous studies have suggested that the symptoms and etiology of ASD indeed form such a spectrum [17], seemingly even extending into the general population [18]. Studying ASD as a quantitative trait (and thus also including subclinical traits) rather than as a categorically defined disorder can contribute to a better understanding of the disorder and the potentially contributing biological pathways. An additional advantage of the use of quantitative severity scores in research is that these continuous scores provide more statistical power and allow the application of advanced statistical methods [17]. We found only a single study that previously investigated the relation between a quantitative measure of ASD severity and tuber count, which did not find an association between cortical tuber count or location and overall ASD severity [19]. Furthermore, ASD is characterized by various difficulties that, according to the latest edition of the Diagnostic and Statistical Manual of Mental Disorders (DSM-5) [20], can be divided in two main domains; (1) deficits in social communication and interaction and (2) restricted or repetitive patterns of behavior, interests or activities. The nature of the symptoms in these domains is substantially different and symptom severity is not necessarily equal in both domains, making it plausible that distinct mechanisms in different brain regions may underlie these two different ASD symptom domains. Therefore, it would be useful to not only study overall ASD severity, but also study the association between symptom severity in these two distinct domains and tuber burden and location in TSC patients.

In addition, it is known that TSC is characterized by a wide range of cognitive abilities and it has been shown that the severity of cognitive impairment is related to tuber burden [11, 21–23]. Similarly, an association between intellectual (dis)ability and autism severity has been demonstrated [24]. It is of interest, but still unclear, whether tuber burden is an independent factor in determining autism severity or if cognitive impairment is an important (potentially mediating) determinant in this association.

Most neuroimaging studies in TSC have focused specifically on tuber characteristics and the association with cognitive or behavioral problems. RMLs can be more difficult to detect, and less is known about the contribution of RMLs to the neurocognitive phenotype in TSC. There are data, however, demonstrating that RMLs are associated with intelligence [25, 26], as well as with the severity of autistic traits [26].

In the present study, we aim to investigate the association between cortical dysplasia (i.e. the number and location of cortical tubers and RMLs) and a clinical observational quantitative measure of ASD severity, and to study the role of cognitive functioning in this association. Furthermore, we aim to investigate the specific association of tuber and RML count and location and ASD severity within the two main domains of ASD symptomatology (deficits in social communication and interaction, and restricted or repetitive behaviors).

## Methods

### Participants

The medical records of all pediatric TSC patients in care at the expertise center ENCORE (Erasmus MC-Sophia Children’s Hospital, Rotterdam, the Netherlands) were retrospectively reviewed. In 75 patients, MRI scans of the brain were available for review. Of this sample, 52 patients (24 boys, 28 girls, 2–17 years of age) also had data on ASD severity and cognitive functioning.

### Measures

#### Autism spectrum symptoms

The severity of ASD was assessed using the Autism Diagnostic Observation Scale (ADOS) [27, 28]. All ADOS assessments were performed and scored by a trained, experienced and certified psychologist or psychiatrist.

For our main analyses, a continuous total standardized calibrated severity score (CSS) was calculated, as well as a CSS for the two separate subdomains of the ADOS (social affect (SA) and restricted and repetitive behaviors (RRB) [29–31]), providing an indication of ASD severity relative to the child’s age and expressive language level [31]. The ADOS CSS is a truly continuous measure, covering the entire spectrum of autistic traits. CSS range between 1 and 10, with lower scores indicating no to very little problems, and higher scores indicating severe ASD symptoms. Raw ADOS total scores corresponding to an ADOS classification ‘Non-spectrum’ are distributed across CSS 1–3, ‘ASD’ across CSS 4–5, and ‘Autism’ across CSS 6–10. For descriptive purposes, children were classified as having ASD (autism or the broader ASD phenotype) or not according to the revised ADOS-2 algorithms [27].

#### Cognitive functioning

Cognitive functioning was assessed using different intelligence measures according to best practice standards; in the majority of children (*n* = 32, 62%) this was either the Wechsler Intelligence Scale for Children-III (WISC-III) or Wechsler Preschool and Primary Scale of Intelligence-III (WPPSI-III) [32, 33]. In some children (*n* = 7, 13%) a non-verbal intelligence test was used, which was either the Wechsler Non Verbal scale of ability (WNV) [34] or Snijders-Oomen Nonverbal Intelligence Test (SON-R) [35]. From all intelligence tests full-scale intelligence quotients (TIQs) were used. For children who were at the floor of their age-appropriate standardized scores, a developmental quotient (DQ) was calculated (developmental age/chronological age × 100) [36]. Like IQ scores, a DQ of 100 is considered the mean. In a number of children (*n* = 13, 25.0%) no formal intelligence test could be performed due to a young (developmental) age. In these children the cognitive developmental age was evaluated using one of the Bayley Scales of Infant and Toddler Development (BSID-II or Bayley-III [37, 38]) or the Vineland Screener [39]. Again, we used the estimated cognitive developmental age to calculate a DQ, according to the formula provided above.

#### Neuroimaging

Brain MRI scans were made on a 1.5 Tesla General Electric scanner. For children with more than one available MRI, the MRI closest in time to the ADOS assessment was selected. All MRI scans were visually inspected by two trained medical students, and re-assessed by a pediatric neuroradiologist and a pediatric neurologist. A protocol for data collection was developed in which fluid-attenuated inversion recovery (FLAIR) images were used to assess the number and location of tubers and RMLs. If FLAIR images were not present, or for clarification purposes, T2-weighted images were used.

### Statistical analysis

Data were analyzed in IBM SPSS Statistics version 21 [40]. Associations between the various variables of interest were studied calculating Pearson correlation coefficients. To investigate the association between ASD severity and total tuber and RML count, linear regression analyses were performed. When these yielded significant results, post hoc analyses were performed, assessing the associations in the separate lobes of the brain. A Bonferroni correction was applied to correct for multiple testing. Because of the considerable intercorrelations between the number of tubers or RMLs in the separate lobes (ranging between 0.63–0.78 and 0.32–0.51, respectively), we first calculated the effective number of tests and adjusted the Bonferroni correction accordingly to account for this lack of independence [41]. The calculation yielded an effective number of 2.98 tests for the tuber analyses and 3.57 tests for the RML analyses. In all tables, both uncorrected as well as Bonferroni corrected (*p*_corr_) *p*-values are provided, as well as *β* and adjusted *R* ^2^ effect size measures. Supplementary linear regression analyses were performed studying the association of ASD severity with the number of cystic and calcified tubers. We also performed supplementary *t* tests and logistic regression analyses, using the categorical ADOS classification instead of the continuous severity scores.

To assess whether IQ/DQ was a mediator in the association between ASD severity and tuber or RML count, formal mediation analyses were performed using the ‘PROCESS’ macro for SPSS, version 2.15 (http://www.afhayes.com/) with bias-corrected bootstrapping using 1000 replications [42]. For the mediation analyses, effect sizes are reported as *κ* ^2^, with values of 0.01, 0.09 and 0.25 considered as small, medium and large respectively [43]. Finally, supplementary mediation analyses were performed to study the role of epilepsy in the association between ASD severity and tuber or RML burden.

## Results

### Patient characteristics

In total, the data of 52 TSC patients (24 boys, 28 girls) were included. The mean age at time of the MRI was 7.0 years (range 0–17) and the mean age at time of the ADOS assessment was 8.8 years (range 2–17). The total number of tubers ranged between 0 and 81 and the total number of RMLs between 0 and 37. The largest number of tubers and RMLs were located in the frontal lobes. According to the ADOS, a total number of 25 children (48.1%) met the criteria for an ASD classification. Additional patient characteristics are shown in Table 1.

**Table 1:** Patient characteristics

	*n* (%)	Mean (SD)	Min–Max
Gender, male	24 (46.2)		
Age in years			
During MRI		7.0 (3.9)	0–17
During ADOS		8.8 (4.2)	2–17
Age difference in years between ADOS-MRI		1.8 (2.9)	0–14
Mutation			
TSC1	14 (26.9)		
TSC2	36 (69.2)		
No mutation identified	1 (1.9)		
Not tested	1 (1.9)		
Epilepsy			
Present, yes	46 (88.5)		
West syndrome, yes (from *n* = 46)	16 (34.8)		
Age of onset, months (from *n* = 46)		15.4 (18.6)	1–91
Tubers			
Present, yes	51 (98.1)		
Number			
Total		27.5 (20.2)	0–81
Frontal lobe		16.0 (12.1)	0–54
Parietal lobe		5.5 (4.6)	0–19
Temporal lobe		3.7 (3.3)	0–12
Occipital lobe		2.4 (2.6)	0–9
Cystic tubers			
Present, yes	19 (36.5)		
Number			
Total		1.85 (3.88)	0–18
Frontal lobe		0.96 (2.21)	0–10
Parietal lobe		0.44 (1.18)	0–7
Temporal lobe		0.33 (0.98)	0–4
Occipital lobe		0.12 (0.32)	0–1
Calcified tubers			
Present, yes	9 (17.3)		
Number			
Total		0.71 (2.52)	0–17
Frontal lobe		0.33 (1.32)	0–9
Parietal lobe		0.15 (0.75)	0–5
Temporal lobe		0.06 (0.31)	0–2
Occipital lobe		0.17 (0.62)	0–3
Radial migration lines			
Present, yes	51 (98.1)		
Number			
Total		16.0 (10.2)	0–37
Frontal lobe		8.2 (5.6)	0–23
Parietal lobe		3.9 (3.3)	0–15
Temporal lobe		3.0 (3.0)	0–12
Occipital lobe		0.9 (1.2)	0–4
IQ/DQ		59.7 (24.5)	8–114
Intellectual disability (IQ/DQ < 70), yes	34 (65.4)		
ADOS module			
Module 1	15 (28.8)		
Module 2	11 (21.2)		
Module 3	18 (34.6)		
Module 4	8 (15.4)		
ADOS classification			
Non-spectrum	27 (51.9)		
Autism spectrum disorder	25 (48.1)		
ADOS calibrated severity score			
Total^a^		4.0 (2.7)	1–10
Social affect domain		4.3 (2.6)	1–10
Restricted and repetitive behaviors domain		4.8 (2.8)	1–10

*n* = 52

*ADOS* Autism Diagnostic Observation Scale, *DQ* developmental quotient, *IQ* intelligence quotient, *MRI* magnetic resonance imaging

^a^Raw ADOS total scores corresponding to an ADOS classification of ‘Autism’ were distributed across calibrated severity scores (CSS) 6–10, ‘ASD’ across 4–5 and ‘Non-spectrum’ across 1–3

In supplementary Table S1 (online resource) the Pearson correlations between all variables of interest are shown. In supplementary Figure S1 (online resource) the distribution of the ADOS total CSS by IQ/DQ is shown, split by ADOS classification.

### Association ASD severity and tuber count

The association between the ADOS total severity score and total tuber count was highly significant (*β* = 0.46, *p* < 0.001), and about 20% of the variance in the ADOS total severity score could be explained by the total number of cortical tubers. Post-hoc analyses assessing the separate lobes of the brain indicated similar results for all lobes (Table 2).

**Table 2:** Association ADOS total calibrated severity score and tuber count

	Model I						Model I + IQ/DQ					
	*B*	95% CI	*β*	*p*	*p*_corr_^a^	$R^{2}_{\text{adj}}$	*B*	95% CI	*β*	*p*	*p*_corr_^a^	$R^{2}_{\text{adj}}$
Total number of tubers	0.06	0.03; 0.09	0.46	**<** **0.001**	–	0.195	0.02	− 0.01; 0.06	0.18	0.188	–	0.356
Frontal lobes	0.09	0.03; 0.15	0.41	**0.002**	**0.007**	0.155	0.03	− 0.04; 0.09	0.11	0.414	1	0.341
Parietal lobes	0.24	0.10; 0.39	0.43	**0.002**	**0.005**	0.166	0.11	− 0.04; 0.25	0.19	0.150	0.447	0.360
Temporal lobes	0.31	0.10; 0.52	0.38	**0.005**	**0.016**	0.129	0.11	− 0.09; 0.31	0.14	0.278	0.829	0.348
Occipital lobes	0.40	0.13; 0.67	0.39	**0.004**	**0.012**	0.136	0.24	− 0.00; 0.47	0.23	0.054	0.160	0.382

*n* = 52

*ADOS* Autism Diagnostic Observation Scale, *IQ* intelligence quotient, *DQ* developmental quotient, $R^{2}_{\text{adj}}$ adjusted R squared model

^a^Multiple testing correction (2.98 effective tests) applied

Significant associations are highlighted in bold font

Because IQ/DQ was significantly related to both the ADOS total severity score and the total number of tubers (as well as to the two separate ADOS subdomain scores and the number of tubers in all separate lobes) (supplementary Table S1, online resource), the analyses were repeated with IQ/DQ added as a covariate. The results of these analyses show that this correction rendered all associations insignificant, indicating that IQ/DQ was an important explanatory variable in the associations (Table 2).

Supplementary analyses studying the association between the categorical ADOS classification and tuber count show similar, but reduced in magnitude, results (supplementary Tables S2 and S3 (online resource).

Next, we studied the association between total tuber count and ASD severity in the two subdomains of the ADOS; social affect (SA) and restricted and repetitive behaviors (RRB) (Table 3). Again, strong associations were found between the total number of tubers and ADOS SA and RRB severity scores (*β* = 0.37, *p* = 0.008 and *β* = 0.49, *p* < 0.001), and 12 and 22% of the variance in respectively SA and RRB severity score was explained by total tuber count.

**Table 3:** Association ADOS subdomain calibrated severity scores and tuber coufnt

	Model I						Model I + IQ/DQ					
	*B*	95% CI	*β*	*p*	*p*_*corr*_^a^	$R^{2}_{\text{adj}}$	*B*	95% CI	*β*	*p*	*p*_*corr*_^a^	$R^{2}_{\text{adj}}$
Total number of tubers												
SA domain CSS	0.05	0.01; 0.08	0.37	**0.008**	–	0.117	0.01	− 0.02; 0.05	0.10	0.497	–	0.262
RRB domain CSS	0.07	0.03; 0.10	0.49	**<0.001**	–	0.224	0.04	0.00; 0.08	0.29	**0.046**	–	0.299
Frontal lobes												
SA domain CSS	0.07	0.01; 0.12	0.32	**0.023**	0.069	0.081	0.00	− 0.06; 0.07	0.02	0.882	1	0.255
RRB domain CSS	0.12	0.06; 0.17	0.49	**<** **0.001**	**0.001**	0.229	0.07	0.00; 0.14	0.30	**0.042**	0.124	0.301
Parietal lobes												
SA domain CSS	0.22	0.08; 0.36	0.40	**0.003**	**0.010**	0.141	0.10	− 0.05; 0.25	0.19	0.169	0.503	0.283
RRB domain CSS	0.22	0.06; 0.38	0.36	**0.008**	**0.025**	0.114	0.09	− 0.08; 0.26	0.15	0.275	0.821	0.257
Temporal lobes												
SA domain CSS	0.20	− 0.01; 0.41	0.26	0.064	0.192	0.048	0.02	− 0.19; 0.23	0.02	0.876	1	0.255
RRB domain CSS	0.37	0.15; 0.58	0.43	**0.001**	**0.004**	0.168	0.21	− 0.02; 0.43	0.25	0.071	0.211	0.288
Occipital lobes												
SA domain CSS	0.34	0.08; 0.61	0.35	**0.012**	**0.036**	0.102	0.20	− 0.05; 0.45	0.20	0.111	0.330	0.293
RRB domain CSS	0.34	0.04; 0.63	0.31	**0.026**	0.079	0.077	0.18	− 0.10; 0.46	0.16	0.201	0.598	0.264

*n* = 52

*ADOS* Autism Diagnostic Observation Scale, *CSS* calibrated severity score, *SA* social affect, *RRB*  restricted and repetitive behaviors, *IQ* intelligence quotient, *DQ* developmental quotient, $R^{2}_{\text{adj}}$ adjusted R squared model

^a^Multiple testing correction (2.98 effective tests) applied

Significant associations are highlighted in bold font

After adding IQ/DQ as a covariate, the total number of tubers only remained significantly associated with the RRB severity score (*β* = 0.29, *p* = 0.046). Post-hoc analyses studying the separate lobes of the brain indicated that this association was mainly driven by tuber count in the frontal lobes (*β* = 0.30, *p* = 0.042, *p*_corr_ = 0.124). An (uncorrected) trend was visible for the association between temporal tuber count and RRB severity (*β* = 0.25, *p* = 0.071, *p*_corr_ = 0.211).

**Fig. 2:**
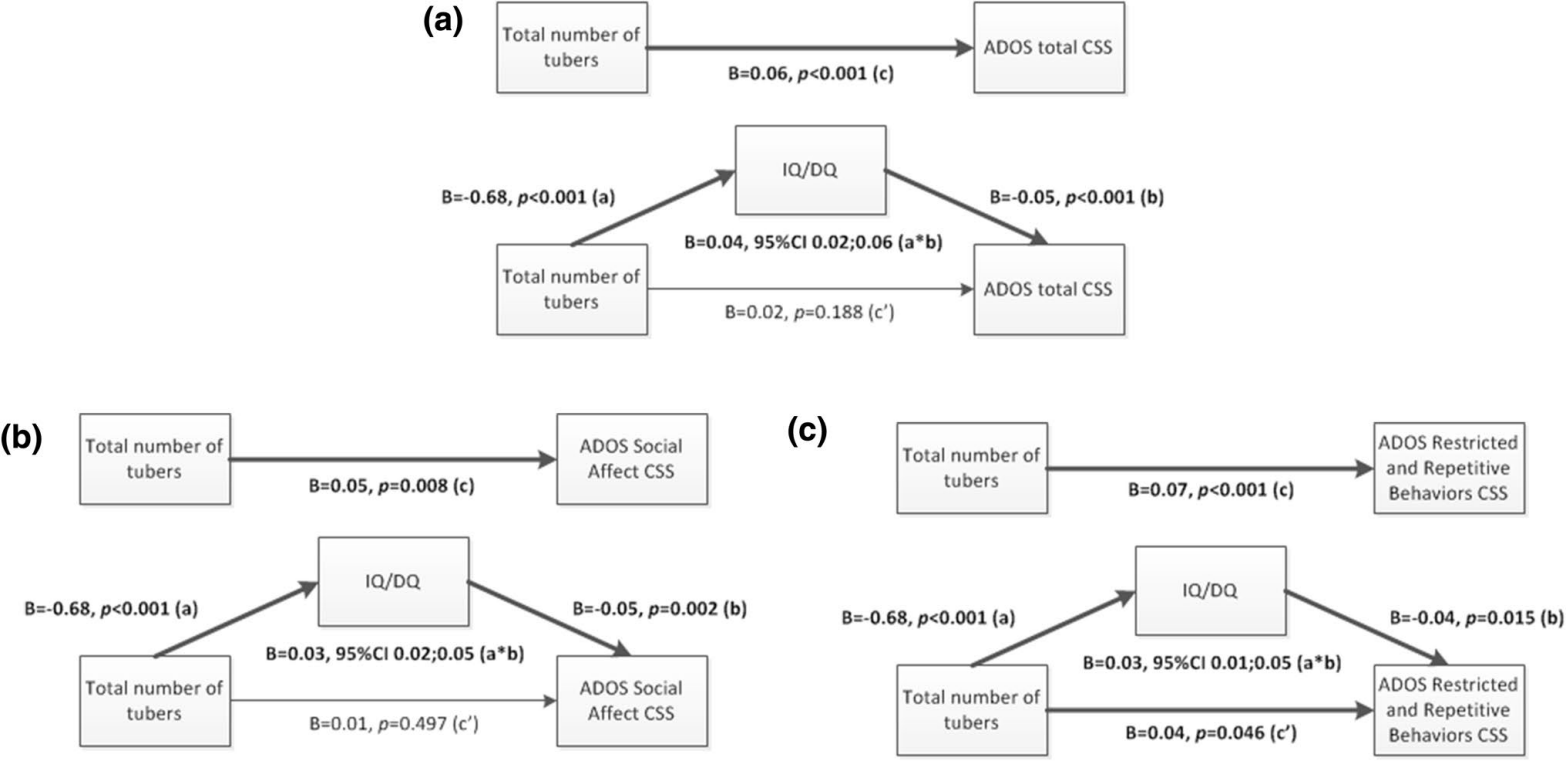
Mediation analyses tuber count, ASD severity score and IQ/DQ. **a** ADOS total severity score, **b** ADOS Social Affect (SA) domain severity score, **c** ADOS Restricted and Repetitive Behaviors (RRB) domain severity score

To formally assess whether IQ/DQ was a mediator in the association between the total number of tubers and the ADOS total severity score, a mediation analysis was performed (Fig. 2, panel a). The mediation analysis showed that the direct effect (c’ path) of total tuber count on the total severity score was insignificant. The indirect (a*b path) effect through IQ/DQ was large and statistically significant (*B* = 0.04, 95% CI = 0.02; 0.06, *κ* ^2^ = 0.263). This implies that the total effect (c path) between total tuber count and the total severity score was fully mediated by IQ/DQ. Post-hoc mediation analyses for the separate lobes were also performed (figures not shown), also showing full mediation by IQ/DQ for all lobes.

Next, mediation analyses were performed studying the role of IQ/DQ in the association between total tuber count and the severity score of the two separate ADOS domains. For the SA domain (Fig. 2, panel b), the direct effect (c’ path) of total tuber count on the SA severity score was insignificant. The indirect effect (a*b path) through IQ/DQ was medium to large and statistically significant (*B* = 0.03, 95% CI = 0.02; 0.05, *κ* ^2^ = 0.242). Again, this means that the total effect (c path) between total tuber count and the SA severity score was fully mediated by IQ/DQ. However, in line with the regression analyses, the mediation analysis performed with the RRB domain (Fig. 2, panel c) showed that although the indirect effect (a*b path) through IQ/DQ was medium to large and significant (*B* = 0.03, 95% CI = 0.01; 0.05, *κ* ^2^ = 0.189), the direct effect (c’ path) of total tuber count on the RRB severity score also remained significant (*B* = 0.04, *p* = 0.046). This means that a direct effect of total tuber count on the RRB severity score was present and that the total effect (c path) was only partly mediated by IQ/DQ. Again, in line with the regression analyses, post hoc analyses of the separate lobes (figures not shown) indicated full mediation by IQ/DQ, with the exception of the frontal lobes. For the frontal lobe association with the RRB score, the indirect effect (a*b path) through IQ/DQ was medium to large and significant (*B* = 0.05, 95% CI = 0.01; 0.09, *κ* ^2^ = 0.188), but the direct effect (c’ path) between frontal lobe tuber count and the RRB severity score remained significant as well (*B* = 0.07, *p* = 0.042), again implying only partial mediation by IQ/DQ.

Because cystic and calcified tubers have been suggested to be more epileptogenic and related to a more severe phenotype [44–47], we additionally studied the associations between the ADOS severity scores and the number of cystic and calcified cortical tubers (supplementary Tables S4 and S5, online resource). These analyses showed that, after correction for IQ/DQ, only the number of calcified tubers was significantly associated with the ADOS total severity score (*β* = 0.27, *p* = 0.020). This association was strongest in, and mainly driven by, the occipital lobe (*β* = 0.33, *p* = 0.003, *p*_corr_ = 0.009) (Table S4). When studying the two ADOS subdomain scores, we found that after correction for IQ/DQ the total number of calcified tubers was significantly related to the RRB subdomain severity score (*β* = 0.32, *p* = 0.010). Post-hoc analyses studying the separate lobes of the brain revealed that this association was strongest in the occipital lobes (*β* = 0.29, *p* = 0.016, *p*_corr_ = 0.046), but also present in the parietal and temporal lobes (*β* = 0.26, *p* = 0.033, *p*_corr_ = 0.094, and *β* = 0.29, *p* = 0.021, *p*_corr_ = 0.060 respectively) (Table S5). Correction for age during MRI did not change these results.

Because studies have suggested that an early onset of epilepsy may have a deleterious influence on early brain development and may act as a risk factor for autism [48], supplementary mediation analyses were performed to study the role of epilepsy in the association between tuber burden and ASD severity (supplementary Figure S2, online resource). The results of these multiple mediation models (that simultaneously included age of epilepsy onset and IQ/DQ as mediators) show that the association between tuber burden and ASD severity was not mediated by age of epilepsy onset (path a2*b2: B = 0.01, 95% CI = − 0.01;0.03), and fully mediated by IQ/DQ (path a1*b1: B = 0.04, 95% CI = 0.02; 0.06). Similar results were found when separately analyzing the two ADOS subdomains.

### Association ASD severity and radial migration line count

A significant association was found between the ADOS total severity score and the total number of radial migration lines (RMLs) (*β* = 0.40, *p* = 0.003), and 15% of the variance in ADOS total severity score was accounted for by the total number of radial migration lines. Post-hoc analyses assessing the separate lobes of the brain indicated significant associations for the parietal lobes and occipital lobes specifically (Table 4).

**Table 4:** Association ADOS total calibrated severity score (CSS) and the number of radial migration lines (RMLs)

	Model I						Model I + IQ/DQ					
	*B*	95% CI	*β*	*p*	*p*_corr_^a^	$R^{2}_{\text{adj}}$	*B*	95% CI	*β*	*p*	*p*_corr_^a^	$R^{2}_{\text{adj}}$
Total number of RMLs	0.11	0.04;0.17	0.40	**0.003**	–	0.146	0.05	− 0.02; 0.11	0.19	0.141	–	0.362
Frontal lobes	0.14	0.02;0.27	0.31	**0.025**	0.090	0.078	0.04	− 0.08; 0.15	0.08	0.505	1	0.338
Parietal lobes	0.28	0.07;0.49	0.35	**0.011**	**0.038**	0.106	0.14	− 0.05; 0.33	0.18	0.140	0.501	0.362
Temporal lobes	0.21	− 0.04; 0.46	0.24	0.094	0.336	0.036	0.10	− 0.11; 0.31	0.11	0.364	1	0.344
Occipital lobes	0.90	0.30;1.50	0.39	**0.004**	**0.015**	0.135	0.65	0.14; 1.16	0.28	**0.013**	**0.047**	0.412

*n* = 52

*ADOS* Autism Diagnostic Observation Scale, *IQ* intelligence quotient, *DQ* developmental quotient, $R^{2}_{\text{adj}}$ adjusted R squared model

^a^Multiple testing correction (3.57 effective tests) applied

Significant associations are highlighted in bold font

Because IQ/DQ was significantly related to both the ADOS total severity score and the total number of RMLs (as well as to the two separate ADOS subdomain scores and the number of RMLs in the frontal and parietal lobes) (supplementary Table S1, online resource), the analyses were repeated with IQ/DQ added as a covariate. After the correction for IQ/DQ only the association for the occipital lobe remained significant (*β* = 0.28, *p* = 0.013, *p*_corr_ = 0.047) (Table 4).

Supplementary analyses studying the association between the categorical ADOS classification and the number of radial migration lines were performed, showing similar effects, but considerably reduced in magnitude and mostly insignificant (supplementary Tables S6 and S7 (online resource).

Next, we studied the association between RML count and ASD severity in the two subdomains of the ADOS; social affect (SA) and restricted and repetitive behaviors (RRB) (Table 5). Again, highly significant associations were found between total RML count and ADOS SA and RRB severity (*β* = 0.41, *p* = 0.002 and *β* = 0.33, *p* = 0.016), and 16% and 9% of the variance in respectively SA and RRB severity score was explained by total RML count.

**Table 5:** Association ADOS subdomain calibrated severity scores (CSS) and the number of radial migration lines (RMLs)

	Model I						Model I + IQ/DQ					
	*B*	95% CI	*β*	*p*	*p*_corr_^a^	$R^{2}_{\text{adj}}$	*B*	95% CI	*β*	*p*	*p*_corr_^a^	$R^{2}_{\text{adj}}$
Total number of RMLs												
SA domain CSS	0.10	0.04;0.17	0.41	**0.002**	–	0.155	0.06	− 0.01; 0.12	0.23	0.080	–	0.300
RRB domain CSS	0.09	0.02; 0.17	0.33	**0.016**	–	0.093	0.04	− 0.04; 0.11	0.14	0.300	–	0.255
Frontal lobes												
SA domain CSS	0.15	0.03; 0.27	0.33	**0.017**	0.061	0.091	0.06	− 0.06; 0.18	0.14	0.296	1	0.271
RRB domain CSS	0.13	− 0.00; 0.27	0.27	0.057	0.203	0.052	0.03	− 0.10; 0.17	0.07	0.608	1	0.243
Parietal lobes												
SA domain CSS	0.27	0.07; 0.47	0.35	**0.010**	**0.037**	0.107	0.15	− 0.04; 0.35	0.20	0.110	0.394	0.293
RRB domain CSS	0.21	− 0.03; 0.44	0.25	0.081	0.288	0.041	0.07	− 0.14; 0.29	0.09	0.504	1	0.246
Temporal lobes												
SA domain CSS	0.20	− 0.04; 0.44	0.23	0.101	0.359	0.034	0.10	− 0.11; 0.32	0.12	0.343	1	0.268
RRB domain CSS	0.24	− 0.03; 0.50	0.25	0.075	0.269	0.043	0.13	− 0.10; 0.37	0.14	0.262	0.935	0.258
Occipital lobes												
SA domain CSS	0.87	0.29; 1.45	0.39	**0.004**	**0.015**	0.137	0.66	0.14; 1.18	0.30	**0.013**	**0.047**	0.343
RRB domain CSS	0.64	− 0.03; 1.31	0.26	0.062	0.222	0.049	0.40	− 0.20; 1.00	0.16	0.186	0.665	0.266

*n* = 52

*ADOS* Autism Diagnostic Observation Scale, *SA* social affect, *RRB* restricted and repetitive behaviors, *IQ* intelligence quotient, *DQ* developmental quotient, $R^{2}_{adj}$ adjusted R squared model

^a^Multiple testing correction (3.57 effective tests) applied

Significant associations are highlighted in bold font

After adding IQ/DQ as a covariate, only the number of RMLs in the occipital lobes remained significantly associated with the SA severity score (*β* = 0.30, *p* = 0.013, *p*_corr_ = 0.047).

**Fig. 3:**
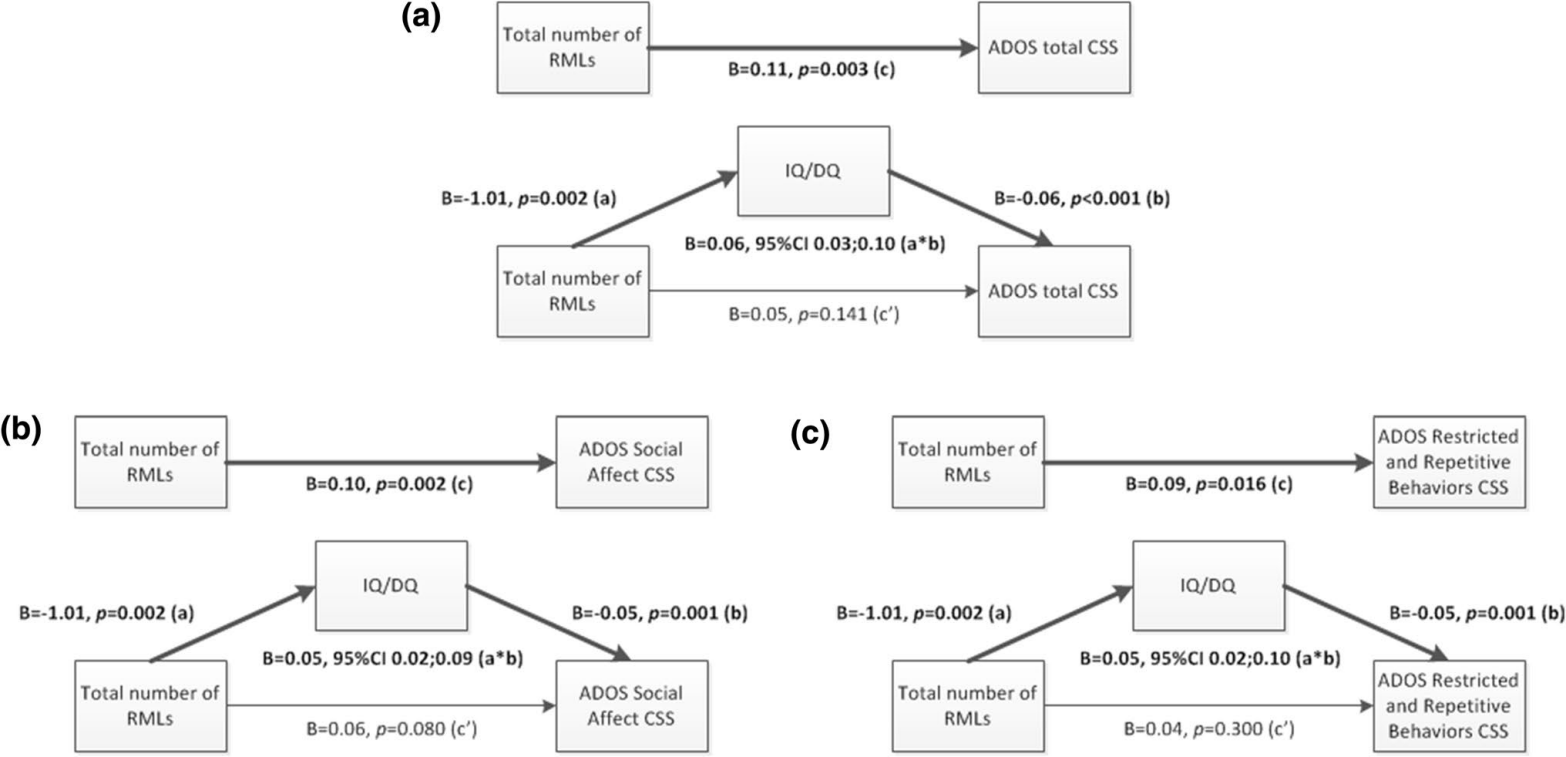
Mediation analyses RML count, ASD severity score and IQ/DQ. **a** ADOS total severity score, **b** ADOS Social Affect (SA) domain severity score, **c** ADOS Restricted and Repetitive Behaviors (RRB) domain severity score

Again, a formal mediation analysis was performed, assessing whether IQ/DQ was a mediator in the association between the total number of RMLs and the ADOS total severity score (Fig. 3, panel a). The mediation analysis showed that the direct effect (c’ path) of total RML count on the total severity score was insignificant. The indirect effect (a*b path) through IQ/DQ was medium to large and statistically significant (*B* = 0.06, 95% CI = 0.03; 0.10, *κ* ^2^ = 0.223). This implies that the total effect (c path) between total RML count and the total severity score was fully mediated by IQ/DQ. Post-hoc mediation analyses for the frontal and parietal lobes were performed as well (figures not shown), also showing full mediation by IQ/DQ. Since IQ/DQ was not significantly related to RML count in the temporal and occipital lobes, the earlier identified association between total number of RMLs in the occipital lobes and the ADOS total severity score could not be mediated by IQ/DQ, implying a direct effect of occipital lobe RML count on the total ADOS severity score.

As with tuber count, the mediation analyses were also performed studying the role of IQ/DQ in the association between total RML count and the severity score of the two separate ADOS subdomains (Fig. 3, panel b and c). For both subdomains, the direct effect (c’ path) of total RML count on the subdomain severity score was insignificant. The indirect effect (a*b path) through IQ/DQ was medium to large and statistically significant (B = 0.05, 95% CI = 0.02; 0.09, *κ* ^2^ = 0.184 for SA domain, and *B* = 0.05, 95% CI = 0.02; 0.10, *κ* ^2^ = 0.190 for RRB domain). This implies that the total effect (c path) between total RML count and the SA and RRB severity scores was fully mediated by IQ/DQ. Post-hoc analyses of the separate lobes (figures not shown) again showed full mediation by IQ/DQ for the frontal and parietal lobes for both the SA and RRB subdomains. Because IQ/DQ was not significantly related to RML count in the temporal and occipital lobes, the earlier found association between RML count in the occipital lobes and the SA severity score could not be mediated by IQ/DQ, implying a direct effect of occipital lobe RML count on the SA severity score.

Finally, supplementary mediation analyses were performed to study the effect of epilepsy on the association between the number of RMLs and ASD severity (supplementary Figure S3, online resource). These multiple mediation models (simultaneously including age of epilepsy onset and IQ/DQ as mediators) show that the association between RML count and ASD severity was not mediated by age of epilepsy onset (path a2*b2: *B* = 0.01, 95% CI = − 0.00;0.03), and was fully mediated by IQ/DQ (path a1*b1: *B* = 0.05, 95% CI = 0.02;0.10). Similar results were obtained when separately analyzing the two ADOS subdomains.

## Discussion

In the current clinical epidemiological study, the association between cortical dysplasia and a quantitative observational measure of ASD severity was studied in a clinical sample of children with TSC. The specificity of the association with the two main subdomains of ASD symptomatology (deficits in social communication and interaction, and restricted or repetitive behaviors) was studied as well. Finally, we focused on the role of cognitive functioning in these associations.

The initial analyses, not corrected for IQ/DQ, showed that total cortical tuber count, as well as tuber count in the separate lobes of the brain, was strongly related to the severity of ASD, visible in both ASD subdomains. However, when IQ/DQ was added as a covariate to the analyses, only total and frontal tuber count remained related to the severity of restricted and repetitive behaviors, although it must be noted that the frontal association did not survive correction for multiple testing and that the relationship may partly arise from the fact that the frontal lobes represent the largest brain area of all lobes. When studying the association between RML count and ASD severity we found similar results, again initially showing strong associations between the number of RMLs and ASD severity, but which were for the most part rendered insignificant when corrected for IQ/DQ, except for the relation between the number of RMLs in the occipital lobes and total and social affect ASD severity. The formal mediation analyses confirmed all results and showed that, indeed, all other initial findings were fully mediated by IQ/DQ.

The results emphasize the importance of taking cognitive functioning into account when studying the relation between brain pathology and ASD in patients with TSC. TSC is often characterized by cognitive impairment, and previous studies have shown that cognitive impairment is strongly related to both brain pathology [11, 21–23] and ASD severity [24], thereby acting as an important confounding (or rather explanatory) factor in this association. However, one should also realize that, regardless of the explanatory role of cognitive functioning, ASD symptoms remain a significant problem in patients with TSC. We found a direct association, regardless of cognitive functioning, between RML count in the occipital lobes and the severity of problems in social communication and interaction. The main function of the occipital lobes is processing visual stimuli, and structural and functional abnormalities in the occipital and occipito-temporal regions have been frequently reported in ASD [49].

One might argue that, next to IQ, epilepsy severity could be an important confounding/explanatory factor in the association between cortical dysplasia and autistic trait severity. A recent study by our group has shown that, in a multivariable model including various epilepsy severity indicators, age of epilepsy onset was the only significant predictor for cognitive functioning later in life [50]. Therefore, we ran supplementary mediation analyses additionally including this variable (as proxy for epilepsy severity) as mediator. The results of these analyses showed that, although there was a significant association between the number of tubers and the age of epilepsy onset, the age of epilepsy onset was not related to any of the ASD severity scores or the number of RMLs, and was no mediator in the associations studied. Because the large majority of our sample (46/52, 88.5%) was using anti-epileptic drugs (AEDs), this sample does not allow us to study the effect of AED use on the described associations. Although the exact effect of AED use on ASD severity remains unclear due to a small number of studies and limited sample sizes, it has been suggested that the use of AEDs may have a beneficial, but most likely very small, effect on ASD severity [51, 52]. If this is indeed the case, the use of AEDs might have attenuated our results. To assist in further elucidating the association between epilepsy and ASD severity in TSC, future studies might not only consider to study the effect of AEDs on ASD severity, but also the effect of other epilepsy indicators such as infantile spasms, epilepsy refractoriness, and current epilepsy status.

The difference in findings between our and other studies (but also between previous studies) can most likely be explained by large differences in methodology, such as (1) different ASD measures (i.e. clinical observational measure vs. screening questionnaire or clinical diagnosis, and continuous severity scores vs. dichotomous diagnostic categories), (2) different ways of defining brain involvement (i.e. tuber/RML count vs. volume or absence/presence of tubers/RMLs), (3) different statistical techniques, (4) the in- or exclusion of confounding variables in the statistical models and (5) participant selection.

A strength and novel aspect of our study is the use of a quantitative measure of ASD severity. Not only does this approach provide a more naturalistic representation of ASD symptoms and more statistical power [17], it also allowed us to study the two different main domains of ASD symptomatology; difficulties in social communication and interaction, and restricted and repetitive behaviors. Another strength of this study is the use of a standardized observational measure of ASD, thereby reducing reporter bias. It must be noted that, although the ADOS is an instrument aiming at measuring autistic traits, it remains unclear whether these traits (especially in non-spectrum patients) truly originate from an ASD predisposition or are caused by other factors that might affect social behavior and restricted or repetitive behaviors. Also, the direction of effect between cortical dysplasia and autistic trait severity remains unclear. Although it seems plausible that the cortical abnormalities have an adverse impact on brain development, consequently leading to more severe ASD symptoms and developmental delay, it might well be that in fact all result from another shared factor.

The stepwise approach and correction for multiple testing in the current study makes it less plausible that findings are false positive, although this cannot be ruled out entirely. Mitigating this concern however, is the modest sample size in which these results were obtained. This relatively small sample size, which is a limitation of our study, might have reduced the power to reveal relatively subtle effects, potentially resulting in an underestimation of effects. It is of great importance that future larger studies attempt to replicate our findings, before any strong conclusions can be made regarding the association between cortical dysplasia and ASD symptom severity. Furthermore, our study employs clinical MRI scans that were made on an 1.5 Tesla scanner. This might have led to limited power to detect RMLs, and made it impossible to accurately retrospectively measure the volume of cortical tubers, thereby preventing us from studying the relation between ASD severity and tuber volume or tuber-brain proportion. Finally, even though the risk of selection bias in our sample is reduced and the generalizability of findings is enhanced by referring all TSC patients within our expertise center for a developmental and psychiatric evaluation (regardless of whether or not the child experiences cognitive or behavioral difficulties), the risk of residual selection bias remains; a first selection takes place when parents decide whether or not to visit the expertise center with their child, and a second selection occurs when parents decide whether or not they want to visit the department of Child and Adolescent Psychiatry/Psychology for a developmental and psychiatric evaluation.

To conclude, our study initially showed strong associations between cortical dysplasia and ASD severity, with children with more cortical tubers and RMLs having more severe ASD symptoms. However, for the majority of these associations, cognitive functioning was identified as an important confounding—or rather explanatory—factor, highlighting the importance of taking cognitive functioning into account when studying the relation between brain pathology and ASD symptomatology. Regardless of cognitive functioning, children with more tubers overall showed more severe restrictive and repetitive behaviors, and children with more RMLs in the occipital lobes specifically showed more difficulties in social communication and interaction. These findings underline the importance of separately studying problems in social communication and interaction on the one hand, and restricted and repetitive behaviors on the other hand.

## Electronic supplementary material

Below is the link to the electronic supplementary material.

Supplementary material 1 (DOCX 344 kb)

## References

[1] Crino PB (2013). Evolving neurobiology of tuberous sclerosis complex. Acta Neuropathol.

[2] Talos DM, Kwiatkowski DJ, Cordero K (2008). Cell-specific alterations of glutamate receptor expression in tuberous sclerosis complex cortical tubers. Ann Neurol.

[3] Crino PB, Trojanowski JQ, Dichter MA, Eberwine J (1996). Embryonic neuronal markers in tuberous sclerosis: single-cell molecular pathology. Neurobiology.

[4] Griffiths PD, Bolton P, Verity C (1998). White matter abnormalities in tuberous sclerosis complex. Acta Radiol.

[5] Curatolo P, Moavero R, de Vries PJ (2015). Neurological and neuropsychiatric aspects of tuberous sclerosis complex. Lancet Neurol.

[6] de Vries PJ, Whittemore VH, Leclezio L (2015). Tuberous Sclerosis Associated Neuropsychiatric Disorders (TAND) and the TAND checklist. Pediatr Neurol.

[7] de Vries PJ, Hunt A, Bolton PF (2007). The psychopathologies of children and adolescents with tuberous sclerosis complex (TSC). Eur Child Adolesc Psychiatry.

[8] Jeste SS, Varcin KJ, Hellemann GS (2016). Symptom profiles of autism spectrum disorder in tuberous sclerosis complex. Neurology.

[9] Bolton PF, Griffiths PD (1997). Association of tuberous sclerosis of temporal lobes with autism and atypical autism. Lancet.

[10] Huang CH, Peng SSF, Weng WC (2015). The relationship of neuroimaging findingsThe relationship of neuroimaging findings and neuropsychiatric comorbidities in children with tuberous sclerosis complex. J Formos Med Assoc.

[11] Bolton PF, Park RJ, Higgins JNP (2002). Neuro-epileptic determinants of autism spectrum disorders in tuberous sclerosis complex. Brain.

[12] Numis AJ, Major P, Montenegro MA (2011). Identification of risk factors for autism spectrum disorders in tuberous sclerosis complex. Neurology.

[13] Curatolo P, Cusmai R, Cortesi F (1991). Neuropsychiatric aspects of tuberous sclerosis. Ann N Y Acad Sci.

[14] Walz NC, Byars AW, Egelhoff JC, Franz DN (2002). Supratentorial tuber location and autism in tuberous sclerosis complex. J Child Neurol.

[15] Asano E, Chugani DC, Muzik O (2001). Autism in tuberous sclerosis complex is related to both cortical and subcortical dysfunction. Neurology.

[16] Hudziak J, Achenbach T, Althoff R, Pine D (2007). A dimensional approach to developmental psychopathology. Int J Methods Psychiatr Res.

[17] Constantino JN (2011). The quantitative nature of autistic social impairment. Pediatr Res.

[18] Blanken LME, Mous SE, Ghassabian A (2015). Cortical morphology in 6- to 10-year old children with autistic traits: a population-based neuroimaging study. Am J Psychiatry.

[19] Weber A, Egelhoff J, McKellop J, Franz D (2000). Autism and the cerebellum: evidence from tuberous sclerosis. J Autism Dev Disord.

[20] American Psychiatric Association (2013) Diagnostic and statistical manual of mental disorders (5th ed.). Am J Psychiatry. doi: 10.1176/appi.books.9780890425596.744053

[21] Kaczorowska M, Jurkiewicz E, Domańska-Pakieła D (2011). Cerebral tuber count and its impact on mental outcome of patients with tuberous sclerosis complex. Epilepsia.

[22] Kassiri J, Snyder TJ, Bhargava R (2011). Cortical tubers, cognition, and epilepsy in tuberous sclerosis. Pediatr Neurol.

[23] O’Callaghan FJK, Harris T, Joinson C (2004). The relation of infantile spasms, tubers, and intelligence in tuberous sclerosis complex. Arch Dis Child.

[24] Hoekstra RA, Happé F, Baron-Cohen S, Ronald A (2009). Association between extreme autistic traits and intellectual disability: insights from a general population twin study. Br J Psychiatry.

[25] Shepherd CW, Houser OW, Gomez MR (1995). MR findings in tuberous sclerosis complex and correlation with seizure development and mental impairment. Am J Neuroradiol.

[26] van Eeghen AM, Terán LO, Johnson J (2013). The neuroanatomical phenotype of tuberous sclerosis complex: focus on radial migration lines. Neuroradiology.

[27] Lord C, Rutter M, DiLavore PC (2012). Autism diagnostic observation schedule, second edition (ADOS-2). Manual (Part I).

[28] Lord C, Rutter M, DiLavore PC, Risi S (1999). Autism diagnostic observation schedule (ADOS). Manual.

[29] Hus V, Gotham K, Lord C (2014). Standardizing ADOS Domain Scores: separating severity of social affect and restricted and repetitive behaviors. J Autism Dev Disord.

[30] Gotham K, Pickles A, Lord C (2009). Standardizing ADOS scores for a measure of severity in autism spectrum disorders. J Autism Dev Disord.

[31] Hus V, Lord C (2014). The Autism diagnostic observation schedule, module 4: revised algorithm and standardized severity scores. J Autism Dev Disord.

[32] Wechsler D (1991). Wechsler intelligence scale for children.

[33] Wechsler D (2002). Wechsler preschool and primary scale of intelligence.

[34] Wechsler D, Naglieri JA (2006). Wechsler nonverbal scale of ability (WNV).

[35] Tellegen P, Winkel M, Wijnberg-Williams B, Laros J (2005). Snijders-Oomen Niet-Verbale Intelligentietest (SON-R) 2 1/2–7: Handleiding en Verantwoording.

[36] van Eeghen AM, Black ME, Pulsifer MB (2012). Genotype and cognitive phenotype of patients with tuberous sclerosis complex. Eur J Hum Genet.

[37] Bayley N (1993). Bayley scales of infant development.

[38] Bayley N (2006). Bayley scales of infant and toddler development.

[39] Scholte E, van Duijn G, Dijkxhoorn Y (2008). Handleiding Vineland Screener 0–6 [Manual Vineland Screener 0–6].

[40] IBM Corp (2012) IBM SPSS Statistics for Windows, version 21.0

[41] Galwey NW (2009). A new measure of the effective number of tests, a practical tool for comparing families of non-independent significance tests. Genet Epidemiol.

[42] Preacher KJ, Hayes AF (2008). Asymptotic and resampling strategies for assessing and comparing indirect effects in multiple mediator models. Behav Res Methods.

[43] Preacher KJ, Kelley K (2011). Effect size measures for mediation models: quantitative strategies for communicating indirect effects. Psychol Methods.

[44] Gallagher A, Madan N, Stemmer-Rachamimov A, Thiele EA (2010). Progressive calcified tuber in a young male with tuberous sclerosis complex. Dev Med Child Neurol.

[45] Koh S, Jayakar P, Dunoyer C (2000). Epilepsy surgery in children with tuberous sclerosis complex: presurgical evaluation and outcome. Epilepsia.

[46] Gallagher A, Grant EP, Madan N (2010). MRI findings reveal three different types of tubers in patients with tuberous sclerosis complex. J Neurol.

[47] Chu-Shore CJ, Major P, Montenegro M, Thiele E (2009). Cyst-like tubers are associated with TSC2 and epilepsy in tuberous sclerosis complex. Neurology.

[48] Curatolo P, Napolioni V, Moavero R (2010). Autism spectrum disorders in tuberous sclerosis: pathogenetic pathways and implications for treatment. J Child Neurol.

[49] O’Connor K, Kirk I (2008). Brief report: atypical social cognition and social behaviours in autism spectrum disorder: A different way of processing rather than an impairment. J Autism Dev Disord.

[50] Overwater IE, Verhaar BJH, Lingsma HF (2017). Interdependence of clinical factors predicting cognition in children with tuberous sclerosis complex. J Neurol.

[51] Hirota T, Veenstra-Vanderweele J, Hollander E, Kishi T (2014). Antiepileptic medications in autism spectrum disorder: a systematic review and meta-analysis. J Autism Dev Disord.

[52] Tuchman R (2004). AEDs and psychotropic drugs in children with autism and epilepsy. Ment Retard Dev Disabil Res Rev.

