# Supplementary material for: Cortical dysplasia and autistic trait severity in children with Tuberous Sclerosis Complex: a clinical epidemiological study
Source: Eur Child Adolesc Psychiatry. 2017 Oct 23;27(6):753–65. doi: 10.1007/s00787-017-1066-z (PMC5973967; doi:10.1007/s00787-017-1066-z)
Supplement: Supplementary file 1 — Supplementary material 1 (DOCX 344 kb) [file 787_2017_1066_MOESM1_ESM.docx]

**Supplementary material**

| **Table S1.** Pearson correlation coefficients main measures. | | | | | | | | | | | | | | |
| --- | --- | --- | --- | --- | --- | --- | --- | --- | --- | --- | --- | --- | --- | --- |
|  |  | 1 | 2 | 3 | 4 | 5 | 6 | 7 | 8 | 9 | 10 | 11 | 12 | 13 |
| 1 | No. of tubers | - |  |  |  |  |  |  |  |  |  |  |  |  |
| 2 | No. of tubers frontal | 0.97** | - |  |  |  |  |  |  |  |  |  |  |  |
| 3 | No. of tubers parietal | 0.84** | 0.73** | - |  |  |  |  |  |  |  |  |  |  |
| 4 | No. of tubers temporal | 0.84** | 0.77** | 0.63** | - |  |  |  |  |  |  |  |  |  |
| 5 | No. of tubers occipital | 0.71** | 0.61** | 0.55** | 0.55** | - |  |  |  |  |  |  |  |  |
| 6 | No. of RMLs | 0.71** | 0.69** | 0.61** | 0.52** | 0.54** | - |  |  |  |  |  |  |  |
| 7 | No. of RMLs frontal | 0.54** | 0.61** | 0.37** | 0.35* | 0.29* | 0.90** | - |  |  |  |  |  |  |
| 8 | No. of RMLs parietal | 0.59** | 0.48** | 0.80** | 0.37** | 0.46** | 0.70** | 0.48** | - |  |  |  |  |  |
| 9 | No. of RMLs temporal | 0.51** | 0.49** | 0.30* | 0.54** | 0.46** | 0.67** | 0.49** | 0.20 | - |  |  |  |  |
| 10 | No. of RMLs occipital | 0.51** | 0.41** | 0.45** | 0.40** | 0.74** | 0.58** | 0.35* | 0.46** | 0.41** | - |  |  |  |
| 11 | ADOS total CSS | 0.46** | 0.41** | 0.43** | 0.38** | 0.39** | 0.40** | 0.31* | 0.35* | 0.24 | 0.39** | - |  |  |
| 12 | ADOS SA domain CSS | 0.37** | 0.32* | 0.40** | 0.26 | 0.35* | 0.41** | 0.33* | 0.35* | 0.23 | 0.39** | 0.92** | - |  |
| 13 | ADOS RRB domain CSS | 0.49** | 0.49** | 0.36** | 0.43** | 0.31* | 0.33* | 0.27 | 0.25 | 0.25 | 0.26 | 0.64** | 0.39** | - |
| 14 | IQ / DQ | -0.56** | -0.56** | -0.47** | -0.45** | -0.31* | -0.42** | -0.40** | -0.322* | -0.22 | -0.20 | -0.60** | -0.53** | -0.52** |
| *Note:* n=52. ADOS=Autism Diagnostic Observation Scale, CSS=calibrated severity score, SA=Social Affect, RMLs=Radial migration lines, RRB=Restricted and Repetitive Behaviors, IQ=intelligence quotient, DQ=developmental quotient. ***p*<0.01, **p*<0.05 | | | | | | | | | | | | | | |

| **Table S2.** T-test comparing individuals without and with an ADOS ASD classification on number of tubers | | | | | | | |
| --- | --- | --- | --- | --- | --- | --- | --- |
|  | **ADOS classification** | **n** | **Mean** | **SD** | **t** | **df** | ***p*** |
| Total number of tubers | non-spectrum | 27 | 19.22 | 16.97 | -3.38 | 50 | **0.001** |
|  | ASD | 25 | 36.52 | 19.88 |  |  |  |
| Frontal lobes | non-spectrum | 27 | 11.44 | 10.06 | -3.04 | 50 | **0.004** |
|  | ASD | 25 | 20.88 | 12.30 |  |  |  |
| Parietal lobes | non-spectrum | 27 | 3.56 | 3.59 | -3.37 | 50 | **0.001** |
|  | ASD | 25 | 7.52 | 4.84 |  |  |  |
| Temporal lobes | non-spectrum | 27 | 2.70 | 2.66 | -2.42 | 50 | **0.019** |
|  | ASD | 25 | 4.84 | 3.66 |  |  |  |
| Occipital lobes | non-spectrum | 27 | 1.56 | 2.31 | -2.53 | 50 | **0.015** |
|  | ASD | 25 | 3.28 | 2.61 |  |  |  |
| Note: ADOS=Autism Diagnostic Observation Scale. ASD=autism spectrum disorder. | | | | | | | |

| **Table S3.** Logistic regression analyses: association ADOS classification (non-spectrum/ASD) and tuber count | | | | | | | | | | | |
| --- | --- | --- | --- | --- | --- | --- | --- | --- | --- | --- | --- |
|  | Model I | | | | |  | Model I + IQ/DQ | | | | |
|  | **B** | **SE** | **OR** | ***p*** | ***p_corr_***^a^ |  | **B** | **SE** | **OR** | ***p*** | ***p_corr_***^a^ |
| Total number of tubers | 0.05 | 0.02 | 1.05 | **0.004** | - |  | 0.02 | 0.02 | 1.02 | 0.224 | - |
| Number of tubers frontal lobes | 0.08 | 0.03 | 1.08 | **0.008** | **0.023** |  | 0.03 | 0.03 | 1.03 | 0.395 | 1 |
| Number of tubers parietal lobes | 0.23 | 0.08 | 1.26 | **0.005** | **0.014** |  | 0.16 | 0.09 | 1.17 | 0.083 | 0.247 |
| Number of tubers temporal lobes | 0.22 | 0.10 | 1.24 | **0.026** | 0.077 |  | 0.07 | 0.11 | 1.07 | 0.536 | 1 |
| Number of tubers occipital lobes | 0.30 | 0.13 | 1.35 | **0.023** | 0.068 |  | 0.17 | 0.13 | 1.19 | 0.187 | 0.559 |
| *Note:* n_tot_=52, n_ASD_=25, n_non-spectrum_=27. ADOS=Autism Diagnostic Observation Scale. ASD=autism spectrum disorder. ^a^Multiple testing correction (2.98 effective tests) applied. | | | | | | | | | | | |

| **Table S4.** Association ADOS total calibrated severity score and cystic/calcified tuber count | | | | | | | | | | | | | |  |
| --- | --- | --- | --- | --- | --- | --- | --- | --- | --- | --- | --- | --- | --- | --- |
|  |  | **Model I** | | | | | |  | **Model I + IQ/DQ** | | | | | |
|  |  | **B** | **95% CI** | **β** | ***p*** | ***p_corr_***^a^ | **R^2^_adj_** |  | **B** | **95% CI** | **β** | ***p*** | ***p_corr_***^a^ | **R^2^_adj_** |
| **Cystic tubers** | |  |  |  |  |  |  |  |  |  |  |  |  |  |
|  | Total number | 0.10 | -0.09;0.30 | 0.15 | 0.281 | - | 0.004 |  | -0.00 | -0.16;0.16 | -0.00 | 0.994 | - | 0.332 |
|  | Frontal lobes | 0.19 | -0.14;0.53 | 0.16 | 0.257 | 0.874 | 0.006 |  | -0.02 | -0.31;0.27 | -0.02 | 0.897 | 1 | 0.333 |
|  | Parietal lobes | 0.33 | -0.31;0.96 | 0.14 | 0.308 | 1 | 0.001 |  | 0.04 | -0.50;0.57 | 0.02 | 0.894 | 1 | 0.333 |
|  | Temporal lobes | 0.26 | -0.50;1.03 | 0.10 | 0.492 | 1 | -0.010 |  | 0.13 | -0.50;0.75 | 0.05 | 0.671 | 1 | 0.335 |
|  | Occipital lobes | -0.75 | -3.08;1.58 | -0.09 | 0.519 | 1 | -0.011 |  | -0.95 | -2.83;0.93 | -0.12 | 0.313 | 1 | 0.346 |
| **Calcified tubers** | |  |  |  |  |  |  |  |  |  |  |  |  |  |
|  | Total number | 0.10 | -0.20;0.39 | 0.09 | 0.523 | - | -0.012 |  | 0.28 | 0.05;0.52 | 0.27 | **0.020** | - | 0.403 |
|  | Frontal lobes | 0.00 | -0.57;0.57 | 0.00 | 1 | 1 | -0.020 |  | 0.40 | -0.07;0.87 | 0.20 | 0.094 | 0.269 | 0.370 |
|  | Parietal lobes | 0.24 | -0.76;1.25 | 0.07 | 0.628 | 1 | -0.015 |  | 0.78 | -0.02;1.59 | 0.22 | 0.057 | 0.163 | 0.380 |
|  | Temporal lobes | -0.62 | -3.07;1.83 | -0.07 | 0.612 | 1 | -0.015 |  | 1.04 | -1.03;3.10 | 0.12 | 0.318 | 0.909 | 0.346 |
|  | Occipital lobes | 1.39 | 0.23;2.55 | 0.32 | **0.020** | 0.057 | 0.086 |  | 1.41 | 0.51;2.32 | 0.33 | **0.003** | **0.009** | 0.445 |
| *Note:* n=52. ADOS=Autism Diagnostic Observation Scale, IQ=intelligence quotient, DQ=developmental quotient. R^2^_adj_=Adjusted R squared model. ^a^Multiple testing correction (3.40 effective tests for cystic tuber analyses, and 2.86 effective tests for calcified tuber analyses) applied. | | | | | | | | | | | | | | |

| **Table S5.** Association ADOS subdomain calibrated severity scores and cystic/calcified tuber count | | | | | | | | |  |  | | | | | |
| --- | --- | --- | --- | --- | --- | --- | --- | --- | --- | --- | --- | --- | --- | --- | --- |
|  |  |  | **Model I** | | | | | |  | **Model I + IQ/DQ** | | | | | |
|  |  |  | **B** | **95% CI** | **β** | ***p*** | ***p_corr_***^a^ | **R^2^_adj_** |  | **B** | **95% CI** | **β** | ***p*** | ***p_corr_***^a^ | **R^2^_adj_** |
| **Cystic tubers** | |  |  |  |  |  |  |  |  |  |  |  |  |  |  |
|  | Total number | SA domain CSS | 0.09 | -0.10;0.27 | 0.13 | 0.354 | - | -0.002 |  | -0.00 | -0.17;0.16 | -0.01 | 0.965 | - | 0.255 |
|  |  | RRB domain CSS | 0.21 | 0.01;0.40 | 0.28 | **0.043** | - | 0.061 |  | 0.12 | -0.07;0.30 | 0.16 | 0.207 | - | 0.263 |
|  | Frontal lobes | SA domain CSS | 0.18 | -0.15;0.50 | 0.15 | 0.275 | 0.935 | 0.004 |  | -0.00 | -0.30;0.29 | -0.00 | 0.993 | 1 | 0.255 |
|  |  | RRB domain CSS | 0.30 | -0.05;0.66 | 0.24 | 0.091 | 0.309 | 0.037 |  | 0.12 | -0.21;0.45 | 0.09 | 0.463 | 1 | 0.247 |
|  | Parietal lobes | SA domain CSS | 0.35 | -0.26;0.96 | 0.16 | 0.252 | 0.857 | 0.007 |  | 0.11 | -0.43;0.65 | 0.05 | 0.693 | 1 | 0.257 |
|  |  | RRB domain CSS | 0.51 | -0.15;1.18 | 0.21 | 0.128 | 0.435 | 0.027 |  | 0.26 | -0.34;0.86 | 0.11 | 0.393 | 1 | 0.250 |
|  | Temporal lobes | SA domain CSS | -0.01 | -0.74;0.74 | -0.00 | 0.990 | 1 | -0.020 |  | -0.12 | -75;0.52 | -0.05 | 0.709 | 1 | 0.257 |
|  |  | RRB domain CSS | 0.85 | 0.07;1.63 | 0.30 | **0.033** | 0.112 | 0.069 |  | 0.73 | 0.06;1.41 | 0.26 | **0.034** | 0.116 | 0.306 |
|  | Occipital lobes | SA domain CSS | -0.54 | -2.79;1.72 | -0.07 | 0.635 | 1 | -0.015 |  | -0.71 | -2.63;1.22 | -0.09 | 0.464 | 1 | 0.263 |
|  |  | RRB domain CSS | 0.64 | -1.84;3.12 | 0.07 | 0.608 | 1 | -0.015 |  | 0.46 | -1.69;2.61 | 0.05 | 0.670 | 1 | 0.242 |
| **Calcified tubers** | |  |  |  |  |  |  |  |  |  |  |  |  |  |  |
|  | Total number | SA domain CSS | 0.06 | -0.23;0.35 | 0.06 | 0.691 | - | -0.017 |  | 0.22 | -0.03;0.47 | 0.21 | 0.084 | - | 0.299 |
|  |  | RRB domain CSS | 0.17 | -0.14;0.49 | 0.16 | 0.271 | - | 0.005 |  | 0.36 | 0.09;0.62 | 0.32 | **0.010** | - | 0.336 |
|  | Frontal lobes | SA domain CSS | -0.01 | -0.56;0.54 | -0.01 | 0.960 | 1 | -0.020 |  | 0.33 | -0.16;0.81 | 0.17 | 0.180 | 0.515 | 0.282 |
|  |  | RRB domain CSS | 0.14 | -0.46;0.75 | 0.07 | 0.633 | 1 | -0.015 |  | 0.53 | -0.00;1.06 | 0.25 | 0.051 | 0.146 | 0.296 |
|  | Parietal lobes | SA domain CSS | 0.16 | -0.81;1.13 | 0.05 | 0.745 | 1 | -0.018 |  | 0.62 | -0.22;1.45 | 0.18 | 0.143 | 0.409 | 0.287 |
|  |  | RRB domain CSS | 0.48 | -0.58;1.54 | 0.13 | 0.366 | 1 | -0.003 |  | 0.99 | 0.09;1.90 | 0.26 | **0.033** | 0.094 | 0.307 |
|  | Temporal lobes | SA domain CSS | -0.81 | -3.17;1.54 | -0.10 | 0.491 | 1 | -0.010 |  | 0.58 | -1.54;2.70 | 0.07 | 0.583 | 1 | 0.259 |
|  |  | RRB domain CSS | 0.97 | -1.62;3.57 | 0.11 | 0.455 | 1 | -0.009 |  | 2.65 | 0.41;4.89 | 0.29 | **0.021** | 0.060 | 0.318 |
|  | Occipital lobes | SA domain CSS | 0.99 | -0.16;2.13 | 0.24 | 0.089 | 0.255 | 0.038 |  | 1.01 | 0.05;1.98 | 0.24 | **0.041** | 0.117 | 0.316 |
|  |  | RRB domain CSS | 1.29 | 0.04;2.54 | 0.28 | **0.043** | 0.123 | 0.061 |  | 1.31 | 0.25;2.37 | 0.29 | **0.016** | **0.046** | 0.324 |
| *Note:* n=52. ADOS=Autism Diagnostic Observation Scale, CSS=calibrated severity score, SA=Social Affect, RRB=Restricted and Repetitive Behaviors, IQ=intelligence quotient, DQ=developmental quotient. R^2^_adj_=Adjusted R squared model. ^a^Multiple testing correction (3.40 effective tests for cystic tuber analyses, and 2.86 effective tests for calcified tuber analyses) applied. | | | | | | | | | | | | | | | |

| **Table S6.** T-test comparing individuals without and with an ADOS ASD classification on number of radial migration lines | | | | | | | |
| --- | --- | --- | --- | --- | --- | --- | --- |
|  | **ADOS classification** | **n** | **Mean** | **SD** | **t** | **df** | ***p*** |
| Total number of RMLs | non-spectrum | 27 | 13.22 | 9.16 | -2.11 | 50 | **0.040** |
|  | ASD | 25 | 19.00 | 10.55 |  |  |  |
| Frontal lobes | non-spectrum | 27 | 6.96 | 5.20 | -1.63 | 50 | 0.110 |
|  | ASD | 25 | 9.52 | 6.12 |  |  |  |
| Parietal lobes | non-spectrum | 27 | 3.15 | 2.64 | -1.81 | 50 | 0.076 |
|  | ASD | 25 | 4.80 | 3.86 |  |  |  |
| Temporal lobes | non-spectrum | 27 | 2.52 | 2.97 | -1.23 | 50 | 0.226 |
|  | ASD | 25 | 3.52 | 2.92 |  |  |  |
| Occipital lobes | non-spectrum | 27 | 0.59 | 0.84 | -1.81 | 50 | 0.077 |
|  | ASD | 25 | 1.16 | 1.37 |  |  |  |
| Note: ADOS=Autism Diagnostic Observation Scale. ASD=autism spectrum disorder. RML=radial migration line. | | | | | | | |

| **Table S7.** Logistic regression analyses: association ADOS classification (non-spectrum/ASD) and radial migration lines | | | | | | | | | | | |
| --- | --- | --- | --- | --- | --- | --- | --- | --- | --- | --- | --- |
|  |  | | | | |  |  | | | | |
|  | **B** | **SE** | **OR** | ***p*** | ***p_corr_***^a^ |  | **B** | **SE** | **OR** | ***p*** | ***p_corr_***^a^ |
| Total number of tubers | 0.06 | 0.03 | 1.06 | **0.046** | - |  | 0.02 | 0.04 | 1.02 | 0.630 | - |
| Number of radial migration lines frontal lobes | 0.08 | 0.05 | 1.09 | 0.113 | 0.404 |  | -0.00 | 0.06 | 1.00 | 0.991 | 1 |
| Number of radial migration lines parietal lobes | 0.16 | 0.10 | 1.18 | 0.088 | 0.313 |  | 0.10 | 0.12 | 1.10 | 0.409 | 1 |
| Number of radial migration lines temporal lobes | 0.12 | 0.10 | 1.13 | 0.226 | 0.808 |  | 0.03 | 0.11 | 1.03 | 0.799 | 1 |
| Number of radial migration lines occipital lobes | 0.47 | 0.27 | 1.60 | 0.087 | 0.310 |  | 0.36 | 0.31 | 1.43 | 0.244 | 0.871 |
| Note: n_tot_=52, n_ASD_=25, n_non-spectrum_=27. ADOS=Autism Diagnostic Observation Scale. ASD=autism spectrum disorder. ^a^Multiple testing correction (3.57 effective tests) applied. | | | | | | | | | | | |


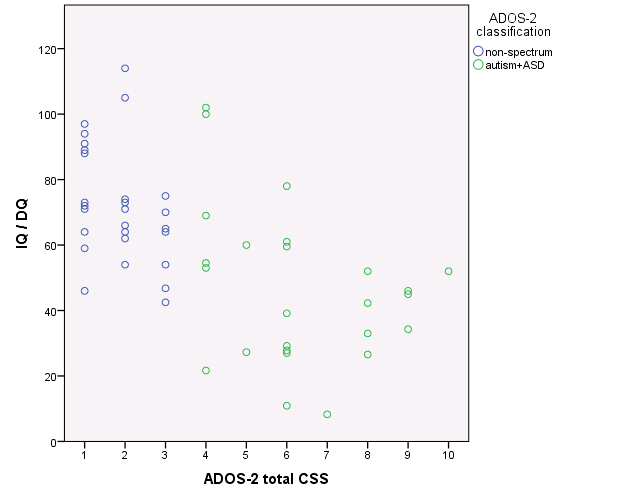


*Figure S1.* Distribution of ADOS total calibrated severity scores (CSS) by IQ/DQ, split by ADOS classification.

*
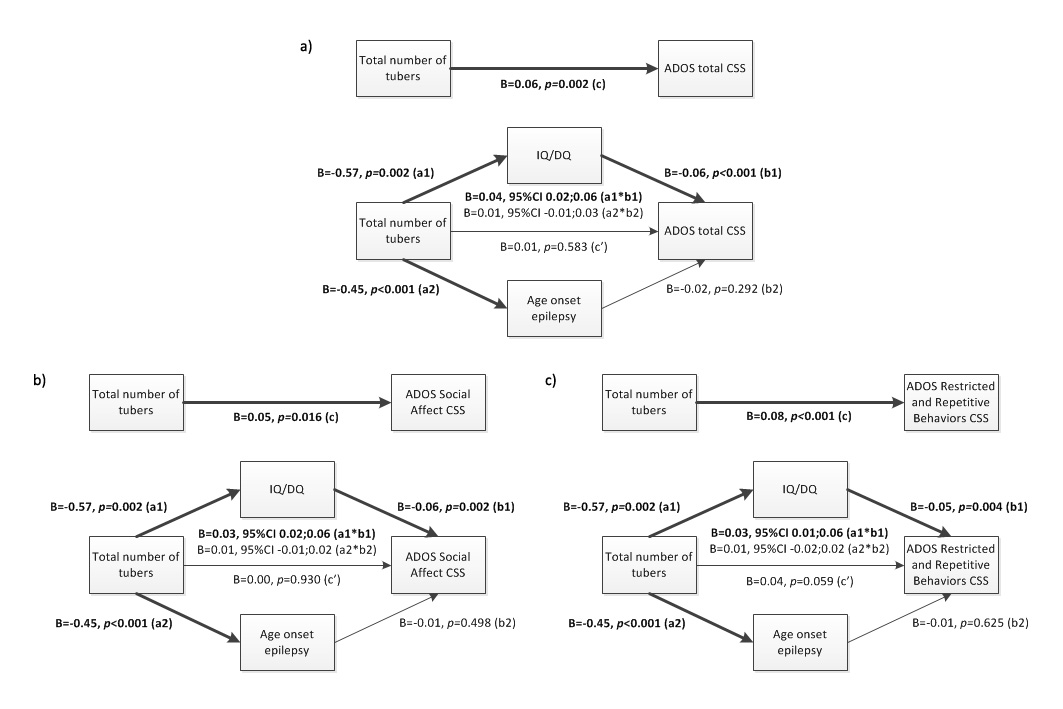
*

*Figure S2.* Mediation analyses tuber count and ASD severity score, with IQ/DQ and age of epilepsy onset as mediators. a) ADOS total severity score, b) ADOS Social Affect (SA) domain severity score, c) ADOS Restricted and Repetitive Behaviors (RRB) domain severity score. *n*=46 (only in children with epilepsy).

*
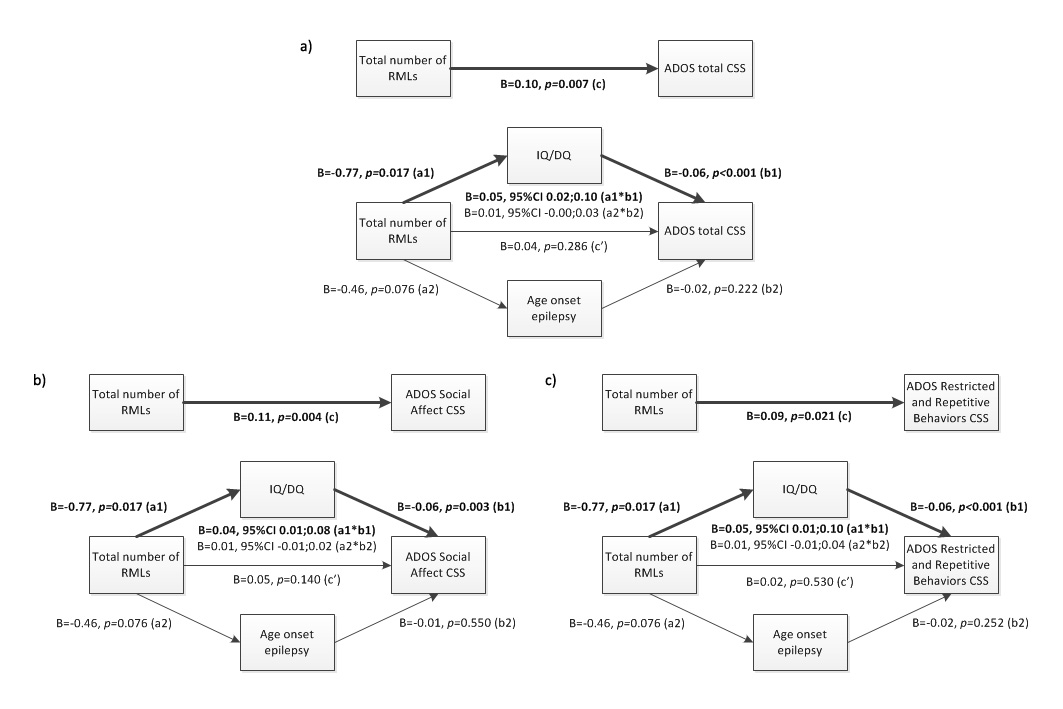
*

*Figure S3.* Mediation analyses RML count and ASD severity score, with IQ/DQ and age of epilepsy onset as mediators. a) ADOS total severity score, b) ADOS Social Affect (SA) domain severity score, c) ADOS Restricted and Repetitive Behaviors (RRB) domain severity score. *n*=46 (only in children with epilepsy).
